# SAFETEL: a pilot randomised controlled trial to assess the feasibility and acceptability of a safety planning and telephone follow-up intervention to reduce suicidal behaviour

**DOI:** 10.1186/s40814-022-01081-5

**Published:** 2022-07-27

**Authors:** Rory C. O’Connor, Susie Smillie, Heather McClelland, Jenna-Marie Lundy, Corinna Stewart, Suzy Syrett, Marcela Gavigan, Alex McConnachie, Bethany Stanley, Michael Smith, Gregory K. Brown, Barbara Stanley, Sharon A. Simpson

**Affiliations:** 1 Suicidal Behaviour Research Laboratory, Institute of Health and Wellbeing, University of Glasgow, Glasgow, UK; 2 Institute of Health and Wellbeing, MRC/CSO Social and Public Health Sciences Unit, University of Glasgow, Glasgow, UK; 3 Institute of Health and Wellbeing, University of Glasgow, Glasgow, UK; 4 Institute of Health and Wellbeing, Robertson Centre for Biostatistics, University of Glasgow, Glasgow, UK; 5 Mental Health Services, NHS Greater Glasgow and Clyde, Glasgow, UK; 6 Perelman School of Medicine, University of Pennsylvania, Philadelphia, PA USA; 7 Department of Psychiatry, Columbia University College of Physicians and Surgeons, New York, USA

**Keywords:** Suicide, Self-harm, Feasibility study, Safety planning, Randomised controlled trial (RCT), Telephone support, Process evaluation

## Abstract

**Background:**

A previous suicide attempt is an important predictor of future suicide. However, there are no evidence-based interventions administered in UK general hospital contexts to reduce suicidal behaviour in patients admitted following a suicide attempt. Consequently, the objective of this pilot randomised controlled trial was to explore whether a safety planning and telephone follow-up intervention (SAFETEL) was feasible and acceptable for individuals treated in hospital following a suicide attempt.

**Methods:**

In this three-phase study with an embedded process evaluation, a safety planning intervention was tailored to the UK context (Phase I), piloted (Phase II, *n* = 32), and tested in a feasibility randomised controlled trial (Phase III). In Phase III, participants were allocated to either the intervention (*n* = 80) or control group (*n* = 40) using telephone randomisation with a 2:1 ratio. The acceptability and feasibility of the trial and intervention procedures were evaluated using both qualitative (interviews and focus groups) and quantitative data. The number of hospital representations of suicidal behaviour was also collected 6 months after study recruitment based on electronic patient records.

**Results:**

Findings indicated that SAFETEL was both acceptable and feasible. Hospital staff reported the intervention fitted and complemented existing services, and patients reported that they favoured the simplicity and person-centred approach of the safety planning intervention.

**Conclusions:**

All progression criteria were met supporting further evaluation of the intervention in a full-scale clinical effectiveness trial.

**Trial registration:**

ISRCT**,** ISRCTN62181241**,** 5/5/2017

## Key messages regarding feasibility

1. What uncertainties existed regarding the feasibility?

- It was not clear whether safety planning with telephone support (SAFETEL) was feasible for use within the UK NHS with patients admitted to hospital following a suicide attempt.

2. What are the key feasibility findings?

- Across 3 study phases including a pilot RCT, we demonstrated that SAFETEL is feasible within the UK NHS context.

3. What are the implications of the feasibility findings for the design of the main study?

- All progression criteria were met supporting further evaluation of the intervention in a full-scale clinical effectiveness trial.

## Introduction

Suicide and self-harm continue to be international health concerns. In the UK, approximately 6000 people die by suicide each year [1], and a previous suicide attempt is a strong predictor of future suicide. In a nationally representative study, it was found that one in nine young adults in the UK had made a suicide attempt at some point in their lives [2], and further evidence indicates that one in 25 people who present at hospital for self-harm die by suicide within the following 5 years [3].

Self-harm is defined here as ‘any act of self-poisoning or self-injury carried out by an individual irrespective of motivation’ [4]. Suicidal behaviour or suicide attempt is also used to describe self-harm where there is evidence of suicidal intent. The prevalence of all forms of self-harm or suicidal behaviour is likely to be underrepresented as not all individuals who self-harm present to hospital, and those who do do not necessarily report that their injuries were self-inflicted [3, 5]. Moreover, despite self-harm and suicidal behaviour being common reasons for presentation to hospital [3], the treatment response is inconsistent. Indeed, the evidence base for interventions administered in general hospital contexts for those patients admitted following a suicide attempt specifically is extremely sparse [6]. This is concerning as individuals are most at risk of re-engaging in life-threatening self-injurious behaviours in the weeks and months following a suicide attempt [7, 8]. A recent meta-analysis of 14 studies concluded that there was evidence for brief suicide prevention interventions, including those with safety planning components, being associated with reduced suicide attempts [6]. Indeed, Stanley and colleagues, in a cohort comparison study of patients presenting to Veterans Affairs (VA) emergency departments for suicide-related concerns, found that safety planning with follow-up telephone support was associated with 45% fewer suicidal behaviours in the subsequent 6 months [9].

However, many gaps in our knowledge remain, including what works for individuals admitted to hospital following a suicide attempt.

Based on the success of Stanley’s a safety planning intervention (SPI) with follow-up telephone support for US veterans presenting to hospital following a suicidal crisis [9], the objective of the current study (SAFETEL) was to address a key gap in UK patient healthcare by exploring the acceptability and feasibility of the SPI with follow-up telephone support within the UK NHS context.

Safety plans are widely used by clinical professionals; however, to date, there has been no randomised controlled trial (RCT) to explore their efficacy in UK general hospital settings for individuals experiencing a suicidal crisis. Consequently, the SAFETEL study involved a 3-phase development and feasibility trial with embedded process evaluation to address the following aims:

1. To adapt/tailor an innovative SPI with follow-up telephone support for use within UK NHS hospital settings
2. To investigate how participants engage with the intervention
3. To assess feasibility and acceptability of the intervention
4. To investigate trial recruitment, retention and other trial processes including data collection
5. To explore the barriers and facilitators to intervention implementation
6. To collect data on readmission to hospital following self-harm in the 6 months following the index suicide attempt to inform the sample size required for a full trial
7. To further develop and test the logic model and theoretical basis of the intervention
8. To assess whether an effectiveness trial is warranted

## Methods

The SAFETEL study was developed in accordance with the Medical Research Council (MRC) guidelines on the development and evaluation of complex interventions [10] and other good practice frameworks [11]. We tested the feasibility and acceptability of both the intervention and the trial evaluation methods, as detailed in our protocol paper [12]. Following best practice, we established an independent Trial Steering Committee who helped us develop progression criteria to directly address the key uncertainties around feasibility and acceptability [13].

**Fig. 1:**
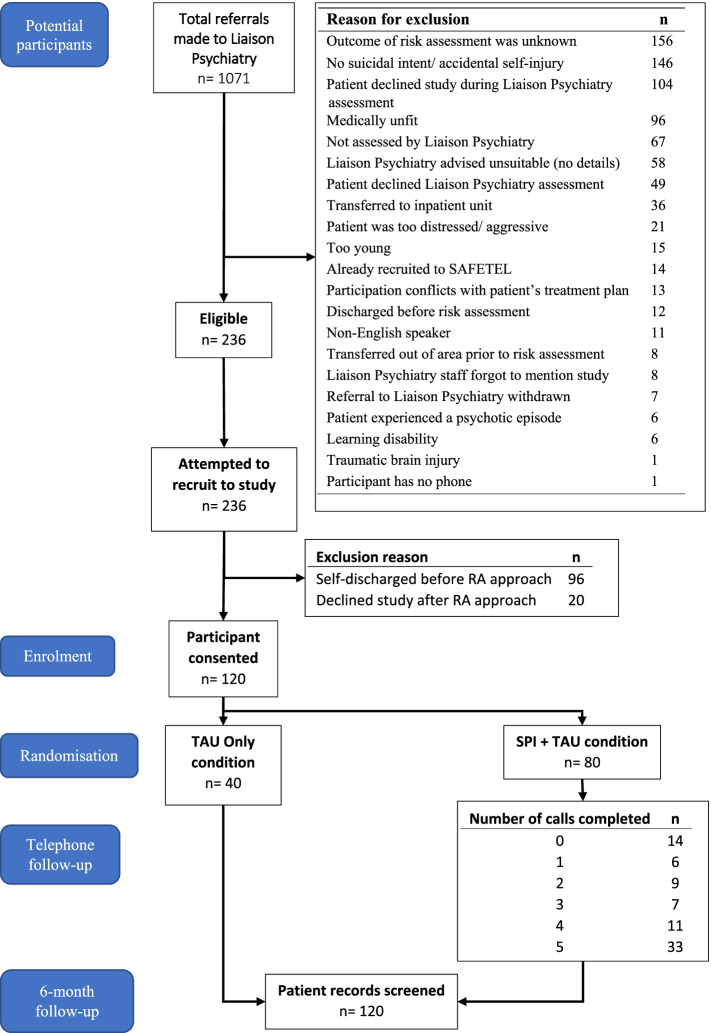
Participant flowchart — Phase III

The study was conducted in three phases:

- Phase I: Consultation and intervention adaptation for the UK context (involving guidance from NHS staff and participants)
- Phase II: Piloting (*N* = 32, with all participants receiving the intervention)
- Phase III: Feasibility RCT (*N* = 120 participants: *N* = 80 SAFETEL intervention + treatment as usual (TAU), *N* = 40 TAU only) (see Fig. 1 Phase III participant flow chart).

Participant recruitment and intervention delivery were conducted independently from the process evaluation. The methods of both the main data collection and process evaluation strategies are described in detail in the protocol paper [12].

### Participant recruitment and eligibility criteria

In Phase I, those with a history of a suicide attempt or with any contact (professionally or personally) with those who have attempted suicide were considered for inclusion. For Phases II and III, participants were eligible for invitation to the SAFETEL study provided they were at least 18 years old and admitted to hospital following a self-harm episode where there was evidence of suicidal intent.

In Phases II and III, following their psychosocial risk assessment by the NHS Liaison Psychiatry team, clinicians based in 4 general hospitals in Scotland made eligible potential participants aware of the study and obtained consent for the research team to provide them with participant information. Full details of the recruitment procedures are provided in the protocol paper [12], and the participant flow for Phase III of the study is outlined in Fig. 1. The East of Scotland Research Ethics Service (EoSRES) approved this study in March 2017 (GN17MH101 Ref: 17/ES/0036).

### Randomisation and blinding

In Phase III, participants were randomly allocated 2:1, to one of two arms via an automated telephone randomisation programme administered by the University of Glasgow’s Robertson Centre for Biostatistics (RCB): SAFETEL safety planning intervention + treatment as usual (SPI + TAU) or treatment as usual only (TAU only). Randomisation was conducted following collection of baseline measures. Randomisation was based on a mixed minimisation algorithm designed to minimise any imbalance with respect of gender, suicide attempt history (‘0–1’ or ‘2 or more’ previous suicide attempts), and hospital site. Following telephone randomisation, both the participant and the intervention researcher were unblinded to the allocated study arm.

### The intervention

Immediately following recruitment and randomisation, in the hospital setting, participants in the SPI + TAU arm engaged in a safety planning intervention (SPI) session. During this session, they completed a Safety Plan collaboratively with the intervention researcher. The Safety Plan comprised of six steps involving person-centred warning signs and helpful responses to an imminent suicidal crisis.

Following completion of the Safety Plan, intervention researchers arranged the first of up to five optional telephone calls (the first call within 72 h of patient discharge from hospital and then weekly thereafter depending on participants’ availability). During follow-up calls, participants’ mood and current level of suicidality were explored, and appropriate escalation procedures followed if participants were found to be at imminent risk of suicide. Use of the Safety Plan and engagement with any supports (personal and professional), since last contact, was explored to help facilitate engagement with both the intervention and other services. Participants were also given the opportunity of updating their Safety Plan. In addition, the call length and number of attempted and completed calls were recorded. Full details of the intervention are published elsewhere [12].

In the TAU only arm (and in addition to the SAFETEL intervention for those in the SPI + TAU condition), participants received treatment as usual which could include referral to one of the following services: primary care, community psychiatric service, third sector services, specialist mental health services, intensive home treatment, outpatient services, inpatient care; or other follow-up services.

### Sample size

As this was a feasibility trial, a formal calculation of the sample size was not required. It was agreed based on previous experience of the research team that a minimum of 30 participants would be adequate in Phase II to inform the refinement of the intervention for Phase III. In Phase III, a sample size of 120 participants (2:1 allocation) was deemed sufficient to explore the feasibility, and acceptability of the intervention. This allowed for an estimation of the outcome event rates for a full trial.

### Data collection

#### Phase I

Focus groups were conducted with hospital staff (*n* = 4, mental health clinicians) and lived experience service users (*N* = 3, people who had experience accessing mental health services) in addition to an interview with a lived experience peer researcher (SS). The interview with hospital staff aimed to identify potential barriers and difficulties implementing the intervention in an acute hospital setting. The focus group with participants with lived experience and the interview with the peer researcher provided feedback about the proposed trial and intervention procedures.

#### Phases II and III: process evaluation

An embedded process evaluation was conducted during Phases II and III in line with the MRC guidance on process evaluation of complex interventions [14]. We developed a process evaluation framework (see Appendix 1: Table 5) which explored fidelity, exposure/adherence, reach and context. In addition, we also assessed recruitment, retention, contamination, and data collection methods to inform a full trial. The process evaluation provided data for assessing the progression criteria. Process evaluation data were collected via quantitative and qualitative methods by intervention researchers and the process evaluation researcher.

#### Recruitment and retention rates

In addition to study recruitment and retention rates, data were collected on the following: the number of eligible potential participants at each hospital site, reasons given for non-referral by NHS Liaison Psychiatry teams (where available), and the number of approaches made by the research team to potential participants regardless of recruitment outcome (see Fig. 1).

#### Intervention fidelity checking

To ensure the intervention was delivered with fidelity, SPI sessions in Phases II and III were audio recorded (with participant consent), and a randomly selected sample (18.75% in Phase II and 30% in Phase III) was assessed using the Safety Planning Intervention Rating Scale [15]. Sessions were rated separately by two trained intervention researchers, ensuring individuals did not check their own session delivery, and disagreement was resolved by discussion. Selected SPI sessions were stratified by recruiting researcher and Phase III recruitment timepoint.

#### Qualitative interviews and focus groups

Semi-structured interviews were conducted with 24 participants in the SPI + TAU arm, seven participants in the TAU only arm, and six NHS Liaison Psychiatry staff. Three focus groups were carried out with eight study researchers. At baseline, study participants were given the option to consent to further contact by the process evaluation researcher at a later stage. Those who consented to this were purposively sampled based on key criteria: gender, engagement with the intervention, age, hospital site, and history of self-harm (see Appendix 2: Table 6). Despite attempts to recruit interview participants who had taken part in the full range of available follow-up telephone calls, those who had attended fewer calls were harder to recruit. Therefore, only one interviewee had not received minimum dose (SPI + 1 follow-up call), and all remaining interview participants (*N* = 23) received three or more follow-up sessions. In Phase II, participants were interviewed within 3 months of recruitment, whereas in Phase III, participants were not interviewed until at least 6 months from their recruitment date to avoid potential contamination of hospital readmission data collection. NHS staff were recruited by emailing and phoning Liaison Psychiatry teams. All those who responded and consented were interviewed. Interviews and focus groups sought to explore participants’, staff, and researchers’ experience of participating in, working alongside, or delivering the study and intervention. Topics included issues around feasibility and acceptability, barriers and facilitators to engaging with or implementing, and broader contextual factors interviewees felt had affected their experience (see Appendix 3: Table 7 for Interview/focus group topic summaries). Transcripts were coded and analysed using the six-phase reflexive thematic analysis approach described by Braun and Clark [16, 17]. Six of the participant interviews (~20%) were double coded by a second researcher to increase reliability. The analysis took both a deductive approach to answer specific questions within the process evaluation framework [14] and an inductive approach to generating themes from the data. Detailed findings from the interviews will be reported elsewhere.

#### Phases II and III: outcome measure feasibility

In Phases II and III, the feasibility of collecting potential outcome measures and moderators for a full trial was explored. These included data collected at baseline and (during Phase III only) at 6 months following the index suicide attempt.

#### Baseline measures

Baseline psychological characteristics were captured using the following measures: Enrichd Social Support Instrument [18], Interpersonal Needs Questionnaire [19], Entrapment Scale [20], Suicide-Related Coping Scale [21], and Columbia-Suicide Severity Rating Scale [22]. See the protocol paper for full details of the measures [12].

#### Six-month post-index episode

In Phase III, individual hospital admission records were reviewed by two intervention researchers, via electronic medical records, to establish whether the participant had re-presented to hospital with an episode of self-harm in the 6 months following their baseline hospitalisation. Where the nature of the hospital re-presentation was unclear (i.e. whether it was self-harm or not or whether there was evidence of suicidal intent), this was discussed between two members of the research team to reach a consensus.

#### Phases II and III: client response forms (CRF)

Intervention researchers recorded all participant (both SPI + TAU and TAU only) data on paper CRFs at baseline and for follow-up calls, which were later entered onto an encrypted electronic database.

#### Treatment as usual

In addition to baseline measures, intervention researchers recorded treatment as usual data for both TAU and SPI + TAU participant groups (see Appendix 4: Table 8).

#### Adherence to intervention

Follow-up call engagement was recorded for each intervention participant. Participants were asked during follow-up calls about the use of their Safety Plan, and this was recorded on the CRF.

### Statistical analysis

Data analysis was conducted by the Robertson Centre for Biostatistics at the University of Glasgow, using R version 3.6.0. Descriptive summaries of participant demographics and scores on the psychometric scales were calculated for Phases II and III participants. Data are summarised descriptively for all participants and by trial arm where appropriate, using counts and percentages for categorical variables and mean and standard deviation (SD), or median and 25th and 75th percentiles (Q1, Q3, respectively, or IQR (interquartile range, IQR)), depending on the distribution of the data. In addition, the primary outcome for a potential full trial (number of readmissions to hospital following self-harm within 6 months of the index suicide attempt) is described by allocated group, and the intervention effect was estimated using negative binomial regression, adjusting for gender and number of previous self-harm episodes. As this was a feasibility study, the statistical significance of any differences between groups was not explored. Incidence rate ratios (IRR), 95% confidence intervals (CI), and *p*-values for the intervention group effect are reported.

### Adverse events

Due to the nature of the study, engagement in suicidal behaviour from participants during the trial was to be expected. Adverse events (AE) and serious adverse event (SAE) were defined in accordance with the National Research Ethics Service and documented for review by the principal investigator (PI) alongside the Trial Steering Committee (TSC). A clinical team member external to the trial administration but a member of the TSC reviewed all AEs and SAEs independently and reported back to the TSC.

## Results

### Refinement of the intervention and trial processes (Phases I and II)

#### Phase I: consultation and intervention adaptation

Feedback from the participants in the lived experience focus group (*n* = 3), and clinical staff (*n* = 4) focus groups, was collated. All of the recommendations identified in Phase I were made to the proposed trial and intervention procedures for Phase II. For a list of these changes, see Appendix 5: Table 9.

#### Phase II: piloting

Across 15 weeks, 32 participants were recruited (for demographic characteristics, psychological characteristics, and follow-up engagement summaries, see Appendix 6: Table 10 and Appendix 7: Table 11). The recruitment, intervention delivery, and follow-up progressed as planned, with no major barriers experienced. Qualitative data from interviews and focus groups (study participants (*n* = 7), NHS staff (*n* = 2), intervention researchers (*n* = 3)), and input from the study peer researcher, informed refinements to the intervention and study methods between Phase II and Phase III. For example, the intervention and study methods were modified in Phase III to address barriers to participant engagement (e.g. losing their Safety Plan after discharge). Changes were largely procedural (e.g. recruitment approach) to improve the participants’ and NHS staff experience. A list of these changes is Appendix 5: Table 9. Feedback from the study peer researcher was extremely beneficial.

### Process evaluation

#### Recruitment and retention (Phases II + III)

Phase II recruitment spanned 15 weeks (24th August–21st November 2017) with recruitment from all four hospital sites. Most (*n* = 27) participants had made a suicide attempt in the past, and over 53% had a history of receiving treatment for mental health difficulties. Across the 33 weeks of Phase III recruitment (31st January 2018–17th September 2018), 1071 patients were referred to Liaison Psychiatry across the 4 hospital sites, presenting with self-harm (irrespective of suicidal intent). Of these referrals, 236 patients were eligible for the study. Some of those who were eligible had been discharged before they could be approached (*n* = 96). Therefore, 140 participants were approached by a member of the research team, of which 85.7% (*n* = 120) consented to take part. No participants withdrew from the study. Completion rate of all items in the psychometric measures was > 99% (*n* = 120).

#### Self-reported participant characteristics

Phase II participant demographics and baseline psychological characteristics are summarised in Appendix 6: Table 10 and Appendix 7: Table 11. Participant characteristics for Phase III are summarised in Table 1. Overall, participants in Phase III had a mean age of 36.4 years old (*SD* = 15.6, range = 18–82): 62.5% identified as female (36.7% male; 0.8%, non-binary) and 96.7% of participants identified as white. Over half (55.8%) reported having been hospitalised at some point in their lives for mental health reasons. A total of 65.8% had a past or present diagnosis of a mood disorder, and 54.2% had received mental health treatment in the last 6 months. The demographic characteristics were similar across the intervention and control arms.

**Table 1:** Phase III participant characteristics (baseline) by intervention group

		All participants (***N*** = 120)	SPI + TAU (***N*** = 80)	TAU only (***N*** = 40)
**Age (mean, SD)**				
		36.4 (15.6)	36.1 (16.1)	37.0 (14.8)
**Gender (** ***n*** **, %)**	Female	75 (62.5)	50 (62.5)	25 (62.5)
	Male	44 (36.7)	29 (36.2)	15 (37.5)
	Non-binary	1 (0.8)	1 (1.2)	0 (0)
**Ethnicity (** ***n*** **, %)**	White	116 (96.7)	78 (97.5)	38 (95.0)
	Asian	3 (2.5)	2 (2.5)	1 (2.5)
	Mixed	1 (0.8)	0 (0)	1 (2.5)
**Education (** ***n*** **, %)**	5.1.1.1. School level qualifications (GCSEs, standard or higher grades, GNVQ, A levels, SVQ levels 1 or 2)	54 (45.0)	39 (48.8)	15 (37.5)
	Further education Advanced GNVQ, HNC or HND, SVQ levels 3 or 4	34 (28.3)	21 (26.3)	13 (32.5)
	Higher education Undergraduate, postgraduate, or PhD degree, SVQ level 5, professional qualification	22 (18.3)	14 (17.5)	8 (20.0)
	No qualifications	9 (7.5)	5 (6.2)	4 (10.0)
	Prefer not to say	1 (0.8)	1 (1.2)	0 (0)
**Lifetime psychiatric history**				
Hospitalised due to mental health problems (*n*, %)	(*n* = 120)	67 (55.8)	40 (50.0)	27 (67.5)
**Lifetime suicidal history**				
Has made a suicide attempt (*n*, %)	(*n* = 120)	113 (94.2)	75 (93.8)	38 (95.0)
Had an interrupted suicide attempt (*n*, %)	(*n* = 120)	56 (46.7)	35 (43.8)	21 (52.5)
Had an aborted suicide attempt (*n*, %)	(*n* = 120)	55 (45.8)	33 (41.2)	22 (55.0)
Engaged in NSSI (*n*, %)	(*n* = 120) Missing data (*n*)	61 (51.3) 1	39 (49.4) 1	22 (55.0) 0
Had a preparatory act taken towards a suicide attempt (*n*, %)	(*n* = 120)	63 (52.5)	39 (48.8)	24 (60.0)

Regarding suicidal history, 94.2% (*n* = 105) of Phase III participants reported they had made at least one prior suicide attempt. Excluding the eight participants who reported “too many suicide attempts to count”, this equated to an average of 3.89 attempts per participant (*SD =* 4.66, range = 1–25, and about one-half also reported a history of self-injury-related behaviours; see Table 1). Phase III participants’ scores on the psychological measures can be found in Appendix 8: Table 12.

#### Intervention fidelity

Analyses of the audio recordings of the SPI sessions, using the Safety Plan Rating Scale [3], yielded an average fidelity score of 91.04% (*SD* = 14.30; *k* = 0.985) in Phase II (across 4 intervention researchers) and 83.91% (*SD*: 14.3; range = 50–100) in Phase III (across 9 intervention researchers) with > 68% of evaluated recordings completing over 80% of the SPI in Phase III.

### Adherence to the intervention

#### Safety Plan intervention session completion

Of the 80 participants allocated to the intervention arm of the study, 78 completed the Safety Plan (97.5%) in hospital, and two completed the baseline measures but declined the Safety Plan and the telephone follow-up. These participants were still randomised as they did not request to withdraw from the study.

#### Follow-up call completion

Total number of participants who completed each timepoint and total number of calls overall are summarised in Tables 2 (Phase II) and 3 (Phase III). Participants could opt out of the follow-up telephone calls at any point.

**Table 2:** Phase II follow-up call engagement by timepoint and total calls completed (*n* = 32)

Timepoint	Call timepoint completed (***n***, %)	Participant expressly opted out of next call (***n***, %)
< 72 h	18 (56.25)	0 (0)
Week 1	24 (75)	0 (0)
Week 2	18 (56.25)	4 (1.25)
Week 3	16 (50)	1 (3.13)
Week 4	18 (56.25)	0 (0)
**Total calls**	**Completed (** ***n*** **, %)**	
0	7 (21.88)	
1	4 (12.5)	
2	2 (6.25)	
3	2 (6.25)	
4	5 (15.4)	
5	12 (37.5)

**Table 3:** Phase III follow-up call engagement (*n* = 80)

Timepoint	Call timepoint completed (***n***, %)	Participant expressly opted out of next call (***n***, %)
< 72 h	52 (65.0)	0 (0)
Week 1	59 (73.8)	5 (8.5)
Week 2	52 (65.0)	2 (3.8)
Week 3	47 (58.8)	17 (36.2)
Week 4	42 (52.5)	NA
**Total calls**	**Completed (** ***n*** **, %)**	
0	14 (17.5)	
1	6 (7.5)	
2	9 (11.3)	
3	7 (8.8)	
4	11 (13.8)	
5	33 (41.3)	

Of Phase III, 82.5% (*n* = 66) of participants in the intervention group achieved the minimum dose for the intervention, defined a priori in collaboration with the independent Trial Steering Committee as the creation of the Safety Plan and at least one follow-up call at any given point. A total of 41% of participants completed all follow-up calls offered to them, while 14 participants (17.5%) did not complete any follow-up telephone calls (see Table 3). Participants were most likely to complete the second call (week 1, 73.8%, *n* = 59) and were most likely to expressly opt out at the fourth call (week 3, 36.2%, *n* = 17). Compared to those who engaged with follow-up calls, those who did not complete any calls were more likely to be male, heterosexual, unemployed, and educated to a high school level of qualification.

#### Safety Plan use (Phase III)

During the telephone calls, 81% of participants discussed the content of their Safety Plan with the intervention researcher and reflected on the relevance of any people and activities they had listed on their Safety Plan. A total of 59% (*n* = 46) of the 78 intervention group participants who made a Safety Plan said they had used it at least once since baseline. Participants were most likely to report using their Safety Plan between their second and third follow-up calls, approximately 1–2 weeks after their recruitment to the study. A total of 47.4% of participants who created a Safety Plan made changes during the follow-up call phase, most commonly during their second follow-up call (21.8%, *n* = 17). Across all calls, the two most commonly changed steps were steps 2 (‘Things I can do to take my mind off my problems without contacting another person’, *n* = 19) and 4 (‘People in their personal lives to contact for support’, *n* = 18).

### Feasibility and acceptability of the intervention

#### Study participant interviews

Participants’ initial motivating factors for agreeing to take part in the study fell into two main areas, and often both were reported: seeking to help others by contributing to research and a desire to have support to improve their own mental health. The perception of the study as ‘not NHS’ or ‘not clinical’ was reported by some participants to be a key motivating factor with some relating this specifically to negative service use experiences in the past. Several participants reported that they felt the timing of the intervention being offered in hospital was key to their agreement to take part. A sense of immediacy experienced in the hospital situation prompted an agreement that might not have been forthcoming in a different context.

I was at that point where I was willing to take any help given to me PE021

Feeling that the particular style of intervention (Safety Plan and telephone calls) would be of benefit to them was frequently reported by participants as a strong motivator for agreeing to take part in the study. Feeling the ongoing benefit of using the Safety Plan and receiving the telephone calls motivated ongoing engagement. However, for some of those participants whose strongest motivation had been to help others continued to engage without feeling that they were gleaning any benefit from this themselves.

The only reason I’m actually still doing this call and everything is because I want there to be a change, and I want people to have the support. PE002

Participants described features of the intervention style that they found to be important facilitators of initial and ongoing engagement. Most frequently, these centred around the level of flexibility, simplicity, and practicality that the intervention offered and where this suited individuals’ needs particularly well.

Everything was just there for you, on one sheet of paper, or on your phone, you could have it with you all the time, if you felt you needed it … I’ve never felt it was difficult to use. I thought the way it was laid out was really good, had all the information there. The stress is kind of off you, as soon as you got that, a lot of it. PE011

Barriers existed where individuals’ specific context made engagement and implementation more challenging and where there was a personal preference for alternative therapeutic approaches.

I found it [completing safety plan] very difficult, to be honest, because, like, suggestions for things that maybe don’t necessarily help me a lot, and then I didn’t have a lot of great people that I could, like, rely on as friends, or as someone to talk to, at that time. So while I would be writing down stuff like that, it wasn’t really something that I could realistically do. PE004

Participants frequently acknowledged that certain skills displayed by the research staff delivering the intervention (e.g. active listening, empathy, patience, non-judgement) were important facilitators in encouraging a positive and productive relationship between themselves and the researchers. In addition, a strong recurrent theme from the participant interviews was around perceived investment of intervention researchers – both in terms of being invested in individual participants’ recovery but also in improving mental health on a broader scale.

It very much felt like, like she felt it was, I think she felt she was using my time rather than I was using her time PE009

once I spoke to [intervention researcher] and that I felt comfy, at ease, it was easy … Somebody that you can sit and talk to and get on with, that understands. PE030

Many of the participants interviewed felt that they had benefitted to some degree from receiving the intervention. Participants reported short-term effects as a result of carrying out their Safety Plan steps (e.g. averting or creating a barrier to suicidal thoughts and behaviour). In addition, longer-term impact was reported by participants (e.g. accessing services, improved warning sign awareness). Beyond using the Safety Plan itself, participants also reported finding a benefit from being able to ‘off-load’ or talk about how they were feeling in the days and weeks after their suicide attempt and from the sense of knowing someone was available to support them at this time.

#### Staff interviews: barriers and facilitators to intervention implementation

Data from the research staff and NHS staff described a number of potential barriers to intervention implementation. Researchers described two key issues: the hospital environment and emotional demands. Variation in the level of privacy that could be offered was problematic, and intervention researchers and participants reported similar techniques in managing this (e.g. drawing curtains, keeping voices low). While the intervention research staff were experienced in dealing with both the demands of working in research and the emotional load involved in providing 1-to-1 support for mental health, it was felt that provision for costed regular clinical supervision should be considered for a future full trial to support staff wellbeing and best intervention provision for participants.

Despite the challenges faced by the research staff, NHS staff generally felt that this kind of intervention fits well within the hospital context. In particular, NHS staff felt that having something to offer patients who would otherwise have no immediate support was particularly positive. Staff who were responsible for assessing and discharging patients reported that they employ some similar strategies like exploring existing supports and signposting, as well as (in some cases) providing some follow-up support after discharge. However, there is considerable variability. Level of demand on NHS services and staff and difficulty in managing existing workloads were felt to be significant barriers in considering any future NHS staff involvement in intervention delivery.

#### Programme theory

**Fig. 2:**
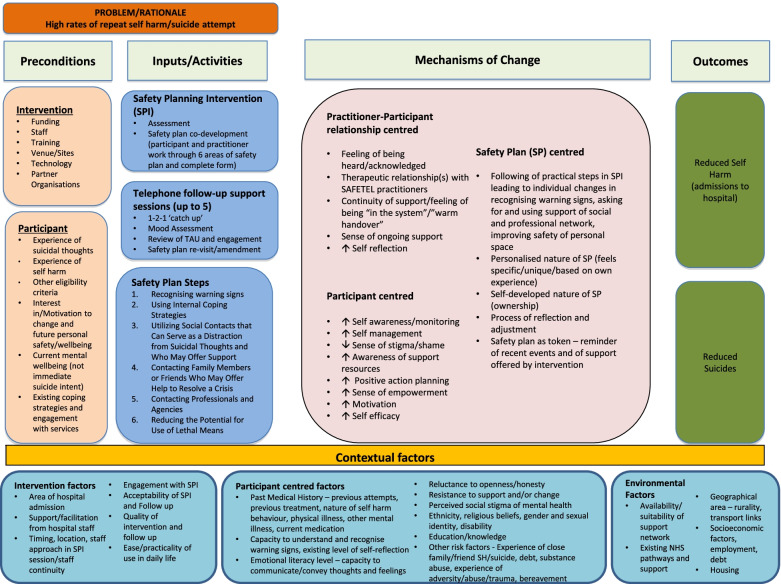
Logic Model

Qualitative data generally supported the intervention programme theory as summarised in the study logic model (Fig. 2). As above, participants emphasised the importance of the therapeutic relationship and the skills of the intervention researchers. Participants also emphasised the importance of ongoing support. Interviews with participants supported, to some extent, all mechanisms of change proposed in the study logic model. For example, there was some evidence that the intervention may have encouraged increased monitoring of triggers and warning signs and increased knowledge of potential positive actions, impacting upon one’s ability to personalise and use Safety Plans and a sense of enhanced self-efficacy as a result. In addition, positive interactions with the intervention researchers increased participants’ motivation to engage with the process of safety planning. Additional potential mechanisms were identified that will be added to the updated programme theory (and reported separately). These include introducing 1-2-1 support in a way that led to increasing openness to accessing talking therapies and other support and normalising seeking and receiving support in both formal and informal ways.

### Feasibility and acceptability of study procedures

#### Qualitative interviews and focus groups

SAFETEL research staff reported that study procedures were generally feasible and acceptable. However, two key issues need to be considered in a full trial: workload and managing communications and relationships with site staff. The same research staff were involved in recruitment (which involved travel time between sites), study administration, and intervention delivery, leading to difficulties in managing schedule and workload. Although we received additional staff resource from the National Health Service Research Scotland Mental Health Research Network to support recruitment, workloads were still high. While overall relationships with hospital sites were positive, the research team reported variation in levels of investment in supporting the study. There were also some issues where referrals were not made to the study despite participants meeting the inclusion criteria. It was not clear whether this was a result of misunderstanding/forgetting inclusion criteria or due to other factors that NHS staff felt made individuals unsuitable for the study. This may have had an impact on the number of referrals and affected the extent to which the referrals were representative of the broader population.

NHS staff reported that accommodating recruitment at their site and the associated presence of intervention research staff was feasible and acceptable. The NHS staff who were interviewed reported generally positive interactions with the research team and did not feel that accommodating study recruitment had put any unfeasible or unacceptable burdens on, or disruptions to, existing hospital practices. However, they noted that workload issues could be a barrier to NHS staff assisting with recruitment in a future trial. There was variability in terms of minor alterations that staff felt could have made the referral process smoother (e.g. research staff attending NHS team meetings). It was also felt that some of the intervention processes could have been tailored to the hospital needs/existing processes, and that this would facilitate good communication between NHS and research staff.

Overall, the majority of feedback provided by intervention arm participants was positive about both the experience of taking part in the study and the intervention, regardless of whether they felt it had been of direct benefit to them. TAU only participants also reported a positive experience with the study, some suggesting they had found the assessment itself beneficial; however, they also reported negative feelings and reactions to being randomised to the TAU only condition.

As noted above, some of the participant and staff feedback was implemented during the trial. Suggestions for more significant alterations were included in a study report to the Trial Steering Committee as recommendations for a future full trial (see Appendix 5: Table 9).

### Treatment as usual (TAU)

**Fig. 3:**
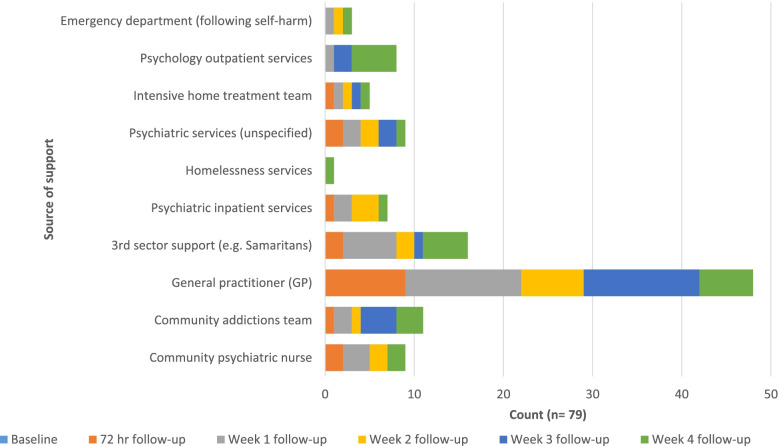
SPI + TAU post-discharge treatment engagement during follow-up call intervention

All participants in the study were offered TAU (i.e. any services offered to them by the NHS staff), and inspection of the baseline referral and self-referral recommendations shows that participants were offered similar options in both study arms (Appendix 4: Table 8). Participants in the intervention arm were asked to provide information about any engagement with services post-discharge from hospital. Figure 3 illustrates that GPs were the most used resource for participants after hospital discharge. Those who engaged with other services (e.g. psychiatry) within 1 week of discharge were typically already being seen by these services prior to hospitalisation. Third sector engagement was highest during week 1 and tended to be a one-off contact.

### Contamination: interview and focus group data

Most participants had not completed or seen something similar to the Safety Plan structure before; however, some had discussed plans of how to keep safe during suicidal crisis with other services or had completed plans in the past that they thought were similar to the Safety Plan used in this study (e.g. ‘Wellness Recovery Action Plans’). None of the participants who had prior experience with safety plans, or similar, reported receiving follow-up support with implementing those. Two participants who were interviewed in the TAU only condition reported investigating safety planning strategies on their own, searching on the Internet, and completing safety plans independently. Participants who were interviewed in the TAU condition did not report receiving any materials from, or knowing anyone in the SPI+TAU condition, nor vice versa.

### Outcome measure feasibility (Phase III)

#### Baseline

Almost 100% (99.99%) of all baseline measures were completed by the 120 participants. Scores on all measures were similar across both groups of participants (see Appendix 8: Table 12). No problems were reported regarding completing the battery of measures in either arm of the study.

#### Six-month post-index admission

Readmission to hospital following self-harm in the 6 months after the index admission was recorded for each participant (see Table 4). Six-month follow-up data were obtained for all 120 participants; 118 records were retrieved using electronic patient records accessed via encrypted NHS computers. The remaining two participants were registered outside of the study NHS health board area and were retrieved by contacting participants’ GPs upon participant consent at baseline.

**Table 4:** Phase III 6-month hospital presentation follow-up

Variable			All participants (*N* = 120)	SPI + TAU (*N* = 80)	TAU only (*N* = 40)	Intervention group effect IRR (95% *CI*)
Number of representations for self-harm	*n* (%)	0	75 (62.5%)	49 (61.3%)	26 (65.0%)	1.10 (0.48, 2.50), *p* = 0.830
		1	24 (20.0%)	16 (20.0%)	8 (20.0%)	
		2	6 (5.0%)	6 (7.5%)	0 (0%)	
		3	5 (4.2%)	2 (2.5%)	3 (7.5%)	
		≥ 4	10 (8.3%)	7 (8.8%)	3 (7.5%)	
Re-presented for self-harm (*n* = 45)	Median (IQR)		1 (1, 3)	1 (1, 3)	1 (1, 3)	
	Mean (SD)		4.2 (6.5)	3.9 (5.7)	4.9 (8.3)	
	Min., max.		1, 30	1, 24	1, 30	
Number of re-presentations to hospital for a suicide attempt	*n* (%)	0	91 (75.8%)	60 (75.0%)	31 (78.0%)	1.10 (0.43, 2.79), *p* = 0.849
		1	17 (14.2%)	11 (13.8%)	6 (15.0%)	
		2	4 (3.0%)	3 (3.8%)	1 (2.5%)	
		3	3 (2.5%)	3 (3.8%)	0 (0%)	
		≥ 4	5 (4.2%)	3 (3.8%)	2 (5.0%)	
Re-presented to hospital for a suicide attempt (*n* = 29)	Median (IQR)		1 (1, 3)	1 (1, 3)	1 (1, 2)	
	Mean (SD)		2.3 (2.5)	2.4 (2.4)	2.3 (2.7)	
	Min., max.		1, 11	1, 11	1, 9	
Duration between index admission and first re-presentation to hospital for self-harm (days)	Median (IQR)		36 (12, 83)	42 (12, 100)	30 (13, 62)	
	Missing (*n*)		3	2	1	

#### Outcome assessment: 6-month follow-up

The study was not designed or powered to calculate effect sizes. However, the number of participants re-presenting to hospital with self-harm, the frequency of these re-presentations, suicide attempt, and time between baseline and first re-presentation are presented in Table 4. There were no significant differences in the outcomes between those in the TAU vs intervention groups.

### Adverse events

Given the nature of the sample, we anticipated re-presentations to hospital during the study engagement period. AEs and SAEs were only captured from participants who were engaged with the follow-up call intervention; other hospital representations were captured when checking hospital records at the end of participant recruitment phase. During Phase II, three individual SAEs were self-reported by three participants. Forty individual SAEs (from 16 individual participants) occurred during Phase III. Two patients accounted for 6 and 7 SAEs each during the 4-week follow-up phone call period. Of the 40 individual SAEs, 29 incidents (73%) were self-reported by the participants to the research team during one of the weekly telephone follow-up time points. Most SAE incidences were treated in outpatient wards of emergency departments where patients were discharged the same day.

Over the course of Phase III, two participants died; one participant was referred to the study in error, with the extent of their injuries becoming apparent after risk assessment by Liaison Psychiatry and recruitment by intervention researcher staff. Another participant died due to a secondary health complication while re-admitted to a hospital as an inpatient several weeks after baseline recruitment. Neither deaths were related to study participation.

### Progression criteria

A set of eight progression criteria for the SAFETEL study was established in collaboration with the independent Trial Steering Committee (TSC) to determine whether a full RCT was warranted (see Appendix 9: Table 13). All progression criteria for this study were met; therefore, the findings suggest that the SAFETEL study and intervention procedures are acceptable and feasible.

## Discussion

This study is the first in the UK to explore the feasibility and acceptability of a safety planning and telephone follow-up intervention to reduce suicidal behaviour within UK NHS hospital settings. Following best practice [10, 13, 14, 17, 23] and adopting a systematic approach, across three study phases, we refined and tested a SPI with follow-up telephone support that had been previously developed for use with veterans in the USA [9]. Overall, the study findings were positive, indicating that the intervention and trial methods were feasible and acceptable. Qualitative data generally supported the intervention programme theory, and all progression criteria outlined in the study aims and agreed in collaboration with the TSC were met. The seven progression criteria which were rated using traffic light cutoffs were judged as green. The eighth criterion, which explored whether barriers and challenges to implementation, and adherence to the intervention, were planned for and were surmountable, was deemed as acceptable. Based on these findings, an effectiveness trial is therefore warranted. A type 2 hybrid effectiveness-implementation design might be most appropriate as this has the dual focus on clinical effectiveness and implementation outcomes [24]. These findings add to the growing evidence base supporting the utility of brief contact interventions including safety planning type interventions [25–27].

Most participants engaged with the intervention, with over 80% discussing the content of their Safety Plan with the intervention researchers at follow-up. This engagement included reflections about the people and activities they had cited in their Safety Plan, which contributed to almost half of participants updating their Safety Plan. From a suicide prevention perspective, it is encouraging that almost 6 in 10 of those who received SAFETEL reported using their Safety Plan at least once during the follow-up period. This finding is noteworthy because greatest risk of repeat suicidal behaviour is within weeks of discharge from hospital following an index suicide attempt [7].

Engagement in the trial seemed to be driven by at least two factors, the altruistic desire to help with research as well as participants’ wish to improve their own mental health. Some participants also indicated they were keen to take part because the intervention was delivered by an independent research team rather than by the NHS, putting this down to negative experiences of service use in the past. This has implications for a full trial which need to be resolved to maximise uptake. If the intervention is delivered by NHS staff, there may be issues with engagement; in addition, staff capacity needs to be considered. The research staff in the current study were able to offer the intervention as an add on to treatment as usual, and as a result, they spent more time with participants than those in the TAU condition. This may have contributed to the more positive experience reported by intervention participants compared to those who received standard care.

The importance of timing was also highlighted, as the intervention was delivered within 24 h of the suicide attempt, and the follow-up telephone support was available within 72 h of hospital discharge. Feedback from those who received the intervention indicated that when someone is in an acute mental health crisis, the sense of immediacy in the hospital environment facilitated their agreement to taking part.

With specific regard to the Safety Plan, the flexibility, simplicity and practicality of the intervention were also cited as important facilitators of initial and ongoing engagement. Although the intervention was manualised, there is flexibility within its delivery to meet the needs of each individual participant. The feedback suggests that participants found it helpful to have their warning signs, distractions and people to contact together on one sheet of paper. However, not having people that they could rely upon to include in the key contacts section was cited as a barrier to using the Safety Plan in practice. Although this was a barrier that we were aware of and sensitive to at the start of trial, this is something that needs to be addressed more closely in a full trial.

The importance of therapeutic skills emerged throughout the interviews. Participants felt valued, listened to and treated with respect and without judgement. Beyond the immediate benefits of the Safety Plan, the importance of the telephone calls was highlighted. They supplemented the safety planning and provided a supportive environment for participants to “off-load” and make sense of their suicide attempt in the weeks after the episode. Unfortunately, we only managed to interview one participant who had not received minimum dose. In a full trial, it may be worth potentially giving more information about a future potential interview at time of recruitment, clarifying that the interview is important for understanding reasons for nonengagement.

In terms of trial procedures, including recruitment, data collection and follow-up, no major barriers were encountered, and the study progressed as planned. Of those approached, almost 70% agreed to take part in the study, and no one withdrew. Although the vast majority of participants received the minimum dose, 17.5% did not complete at least one follow-up telephone call after completing the SPI. Unfortunately, as we were only able to recruit one person who did not receive the minimum dose to the process evaluation, we are limited in what we can say in terms of the reasons. However, from previous studies, this lack of engagement is likely to be for a number of different reasons such as the following: after being discharged from hospital, they did not need any further support, they did not want to receive the calls as they served as a reminder of their suicide attempt, they did not think the calls would be helpful or they were receiving other support already. In an effectiveness-implementation trial, trying to reach these individuals should be considered. The study would have benefited from another dedicated staff member for recruitment and intervention delivery. In practical terms, the limited staff resources and remote location of the research team to the hospitals impacted upon the recruitment rate because by the time patients were approached in hospitals, they were ready to be discharged from hospital and therefore reluctant to take part. Additionally, a further limitation was the capturing of hospital re-presentations; the level of detail of hospital presentation varied considerably between health professionals leading to difficulty in determining the nature of the presentation.

Several practical issues around managing communications with NHS staff, the hospital environment, workload and the emotional demands on the research staff were identified. All were managed but could be optimised in a full-scale trial. Staff workload was high; this was a direct effect of having a ceiling on the grant funding available for the trial and to pay for more staff time. Ideally, as noted above, more research staff, in addition to allocated administrative support, would have aided recruitment and intervention delivery. This would have been beneficial as, inevitably when recruitment is across multiple hospital sites, there was a lot of travel which is time-consuming and distracts from study delivery and implementation. In addition, while it was possible to avail of informal clinical supervision and support throughout the trial, this should be costed and formalised in a full RCT to ensure that this is available to all staff. The NHS staff were very positive about the intervention and trial delivery. However, they recognised that the level of demands on NHS services and staff could be a potential barrier if consideration is being given to NHS staff recruiting or delivering the intervention. As such, a dedicated independent group of staff for recruitment/intervention delivery during a full trial is recommended (a third sector mental health organisation could be considered); this would allow staff sufficient time to approach all potential participants. Although the percentage of our sample who identified as ethnic minority was similar to that for Scotland as a whole (in the last census in Scotland [28], 4% of the Scottish population identified as Asian, African, Caribbean, or Black, Mixed, or other ethnic groups; 3.3% of our Phase III sample were of Asian or Mixed ethnicity), it would be useful to explore whether the intervention needs further tailoring for those from different ethnic minority backgrounds.

In conclusion, the findings of this study support further evaluation of the safety planning and telephone follow-up intervention (SAFETEL) to reduce suicidal behaviour. Indeed, given the encouraging findings from recent meta-analyses of brief acute care suicide prevention and safety planning-type interventions being associated with reduced suicide risk [26, 27], a full trial of SAFETEL is an urgent priority.

## Data Availability

Data and intervention materials will be made available upon reasonable request.

## Appendix 1

**Table 5:** Process evaluation framework for analysis

Evaluation area	Questions
**Fidelity** (The degree to which the intervention was delivered as intended)	• What is the intervention? • Was the intervention (safety plan and follow-up telephone calls) delivered as intended? • Was there consistency in terms of how the intervention was delivered? • What, if any, adaptations were needed to the planned intervention? And were they needed? • What barriers, if any, were there to delivering the intervention in a consistent way? (Safety plan and follow-up telephone calls)
**Exposure** (The extent to which participants received and understood the different elements of the intervention and whether they implemented these as intended. Their satisfaction with the intervention and barriers to receipt and implementation was also considered)	• To what extent did participants take up all potential elements of the full programme of intervention (safety plan and 5 follow-up telephone calls)? • To what degree did participants receive the minimum dose (safety plan and 1 follow-up phone call)? • To what extent was the safety plan completed as intended by the participant? If it was not, what were the reasons for that? • How did participants use the safety plan they had developed? (E.g. frequency of use, practicality — where did they keep it, did they share with others) • To what extent did participants alter or amend their safety plans throughout the course of the intervention? • What elements of the intervention did participants find helpful/unhelpful and why? What elements of the intervention would participants change and why? • What changes, if any, did participants feel that they implemented as a result of taking part in the intervention? • What factors were involved in ongoing engagement with the intervention? • What do participants report were barriers and facilitators to developing the safety plan, engaging with telephone support, and using the safety plan in practice? • What feedback do participants have regarding feasibility and acceptability of the safety plan and follow-up telephone calls?
**Reach** (The extent to which the target audience is reached by the intervention, as well as any ‘spillover’ effects on people not recruited)	• How well does the study sample represent the population of interest? • Did participants report sharing their SP with family or friends? • To what extent did the intervention reach and influence people other than recruited participants?
**Context** (Includes information relating to aspects of the context in which the intervention was delivered, as well as broader context that both practitioner and client were operating within that may influence intervention effectiveness)	• What participant-centred contextual factors influenced engagement with the intervention (safety planning and follow-up calls) and use of the safety plan in practice? • What contextual factors within participants’ day-to-day environment influenced engagement with the intervention (safety planning and follow-up calls) and use of the safety plan in practice? • How did the context in which the intervention was delivered influence engagement with the intervention and use of the safety plan in practice? • Was the safety plan useful in certain circumstances and not in others? • How does the intervention fit in with what is delivered in hospital (how easy was is it to deliver in this setting and does it conflict with anything)? • What were the particular context-related difficulties/issues that arose during the study in delivering the intervention?
**Recruitment and retention**	• How did participants feel about being approached/recruited in hospital setting? • How acceptable were study and intervention procedures to participants? • What motivated study participants to agree to take part? (And what kept them engaged?) • Were there any difficulties in recruitment? • What is the attrition rate overall and by subgroup? I.e. intervention groups and control • What were the reasons for withdrawal?
**Contamination**	• What are the characteristics of other groups or services people are attending or resources they are using — do these provide any elements of the intervention? • Have participants used a safety plan or similar in the past? • Did participants in the TAU (treatment as usual) arm investigate ‘safety plan’ strategy on their own? • Have any of the TAU arm participants seen intervention content from other participants? • How did randomisation to the TAU arm affect participants?

## Appendix 2

**Table 6:** Process evaluation: study participant interviewee characteristics

	Overall study participants (*n* = 152) (*n*, %)		Process evaluation interviewees (*n* = 31) (*n*, %)	
Trial arm (*n*, %)				
SPI + TAU	112	74%	24	77%
TAU only	40	26%	7	23%
Age group				
Age 18–25	43	28%	11	35%
Age 26–40	52	34%	12	39%
Age > 40	57	38%	8	26%
Gender				
Female	94	62%	23	74%
Male	57	38%	8	26%
Non-binary	1	1%	0	0%
History of self-harm with suicidal intent				
0 or 1	42	28%	11	35%
2 or more	110	72%	20	65%
Hospital recruitment site				
H1	26	17%	5	16%
H2	59	39%	10	32%
H3	29	19%	6	19%
H4	38	25%	10	32%
Level of engagement (minimum dose received)				
Intervention arm participants who reached minimum dose (SPI + 1 FU)	91	81%	23	96%
Intervention arm participants who did not receive minimum dose	21	19%	1	4%

## Appendix 3

**Table 7:** Process evaluation interview/focus group topic summary

**Context** • Past experience of mental health services, use of safety plan, or similar • Existing/usual coping strategies • Existing/usual support (formal/informal) **Recruitment** • Acceptability of trial procedures • Motivation to take part/expectations • Suggested improvements/changes acceptability of randomisation **Intervention (SPI + TAU participants only)** • SPI session °Location, timing, setting °Barriers/facilitators °Level of understanding °Expectations of SP use •SAFETEL intervention researcher ° Engagement with researcher — barriers/facilitators ° Acceptability/level of participant comfort ° Helpful/unhelpful aspects • Safety plan use ° Use in practice ° Circumstances of use ° Barriers/facilitators • Follow-up calls ° Facilitators/barriers to engaging ° Acceptability ° Helpful/unhelpful aspects ° Using call — changes to SP/coping strategies • Overall ° Level of support ° Changes implemented ° Comparison to other services/interventions/coping strategies used ° Suggestions for improvement **Since recruitment (3 months in Phase II, 6 months in Phase III)** • Changes made/experienced • Other services or support • Use of Safety Plan or related coping strategies **General** • Helpful or unhelpful aspects of participation • Suggested improvements of other feedback **NHS Liaison Psychiatry staff** • Level of contact with SAFETEL team, knowledge of study • Current context and services available at site, e.g. discharge/referral procedures • Acceptability of trial procedures and fit within hospital context • Acceptability of intervention elements • Feasibility of delivering the intervention • Barriers/facilitators for implementing • Knowledge of other similar interventions/services • Suggestions for improvements/other feedback **SAFETEL researchers delivering intervention** Recruitment • Acceptability, feasibility, and facilitators/barriers affecting the following: ° Identification of potential participants ° Approaching potential participants ° Recruitment procedures: information, consent, baseline data collection, randomisation SPI session • Setting — appropriateness, acceptability, variability • Factors affecting SPI development — barriers/facilitators, strategies used to overcome issues Follow-up calls • Acceptability • Feasibility • Practical changes implemented • Barriers and facilitators to engaging participants • How participants used calls Safety Plan use • Participant engagement with SP • Perceived barriers/facilitators to implementing SP use Participant feedback • Perceived/reported helpful/unhelpful elements of intervention • Perceived/reported barriers/facilitators to engaging with study/intervention • Participant suggestions for improvement • Awareness of past SP use or other similar service use Overall • Other reflections on trial and intervention procedures — positive/negative aspects, facilitators/barriers • Impact on staff — wellbeing, training, support • Perceived impact on participants of study and intervention engagement Suggested adaptations	

## Appendix 4

**Table 8:** Baseline referrals and self-referral recommendations for Phase III total sample group

	SPI + TAU (*N* = 79)		TAU only (*N* = 40)	
	5.1.1.2. *n*	%	5.1.1.3. *n*	%
Primary care	43	54.43%	26	65.00%
Community psychiatric care	31	39.24%	11	27.50%
Third sector referral	9	11.39%	4	10.00%
Specialist mental health service	23	29.11%	10	25.00%
Contact from intensive home treatment team	3	3.80%	3	7.50%
Outpatient services	6	7.59%	4	10.00%
Inpatient services	2	2.53%	1	2.50%
Received contact from other services	44	55.70%	22	55.00%
No further treatment	4	5.06%	1	2.50%

## Appendix 5

**Table 9:** Changes following Phases I, II, and III stakeholder feedback

Phase in which feedback was received	Change
**I**	
	• Participants should be reminded that they do not have to answer any questions that they do not want to; their responses are voluntary
	• Where possible, terminologies used to describe the psychometric assessments should be altered to reduce stigma without compromising the reliability of the measure
	• Staff should remind participants that they were entitled to take breaks whenever they choose, and that researchers should work at the participants’ pace
	• The ‘treatment as usual’ form should be moved to the end of the study, so as to support the movement back to more general discussions as this was felt to be a more natural way to bring discussions to an end. This should be followed by asking participants what they planned to do once they are discharged from hospital
	• Researchers should be reminded to consistently risk assess participants throughout the psychometric assessment completion phase, to ensure that asking the questions does not have a negative effect on participant wellbeing
	• Participants should be provided with envelopes to keep their Safety Plan and other study materials safe and discrete. Participants should also be asked if they would like to take a picture of their safety plan on their phones so that it is visually available to them at all times
**II**	
	• Participants’ study documents (safety plan, consent form, participant information sheet) should be sealed in a green envelope to help it stand out from other hospital discharge documents
	• Researchers should offer additional copies of the Safety Plan to participants following each follow-up call to facilitate the use of the Safety Plan
	• The script for the first follow-up call should be amended to acknowledge that participants may have forgotten aspects of their baseline contribution to the study, including how to use the Safety Plan. This amendment should include offering to explain the function of the safety plan again
	• Researchers should continue to remind participants that they can edit the safety plan at any point, and that it is a dynamic document
	• Researchers should remind participants that others have found the Safety Plan to be of benefit, so we hope that it may also be of personal benefit to them
	• Researchers should check that prospective participants have both been risk assessed and notified about the study so they can indicate if they wish to be approached initially
	• Researchers should intermittently check-in with NHS referral staff to ensure existing operating procedures (e.g. times for telephone calls to check eligibility) continue to be at a suitable time
	• Minor changes to the CRF were suggested, to help clarify TAU (e.g. in-patient vs out-patient psychological support) and mental health diagnoses (e.g. anxiety vs. PTSD)
	• A coping measure (Suicide-Related Coping Scale (SRCS)) to explore coping styles of the participants at baseline should be incorporated into the protocol
**III**	
	• A brief one-page bullet point summary of the content of the PIS (what is involved in the study and participants’ rights) to accompany the PIS should be developed
	• A tutorial video (e.g. using YouTube, Vimeo, with no option for participants to add comments below the video) detailing the Safety PIanning Intervention (SPI), what is involved, level of flexibility, and the parameters of the interventions (what the SPI is and is not) should be developed. This could be sent to participants via text for them to watch in their own time. Researchers should inquire and record whether participants used the video during the follow-up calls
	• It would be useful to examine how participants relate to SPI engagement, perhaps using data already collected in the CRF (e.g. controllability of suicidal thoughts item in CSSRS; ENQ; SCRS) • Consider providing a handwritten (or sticker) reminder of the name and telephone number that will be used to call participants (mobile and desk number) on the green envelopes. This may improve follow-up telephone call engagement, particularly at the initial 72-h call point
	• The first call (within 72 h of baseline assessment) could be considered a ‘touching base’ contact, where participants are informed about the structure of each call, flexibility regarding calls, etc. and invited to review their Safety Plan (may be especially beneficial for those who have difficulty remembering their Safety Plan from baseline in hospital)
	• Research team should determine parameters regarding personalisation of SPI and flexibility of calls (e.g. absolute max. and min. time between calls, number of extra calls offered) as these should be standardised across all sites according to the study protocol
	• The Safety Plan script should be updated to explicitly ask for likelihood of use and barriers at each step. To ensure this, quarterly fidelity checks of Safety Plan recordings using the official Safety Plan Fidelity Monitoring Checklist should be conducted

## Appendix 6

**Table 10:** Participant characteristics (Phase II)

Variable		Mean (SD)/***N*** (%)
**Age (at baseline)** **(mean, SD)**		
	5.1.1.4. *N* = 32	38.88 (14.07)
**Gender (** ***n*** **, %)**		
	Female	17 (53.12)
	Male	15 (46.87)
**Ethnicity (** ***n*** **, %)**		
	White	31 (96.88)
	Asian	5.1.1.5. 1 (3.12)
**Relationship status (** ***n*** **, %)**		
	Single	17 (53.12)
	In a relationship (dating)	2 (6.25)
	Married/civil partnership/common-law marriage/living with partner	8 (25)
	Separated/divorced/dissolved civil partnership	5 (18.75)
**Sexual orientation (** ***n*** **, %)**		
	Heterosexual/straight	25 (78.13)
	Gay/lesbian	3 (9.38)
	Other	1 (3.12)
	Prefer not to say	3 (9.38)
**Living status* (** ***n*** **, %)**		
**Multiple options allowed for this question, i.e. spouse and children*	Living alone	7 (21.88)
	Cohabiting with	
	Spouse, common-law partner, partner, ex-partner	8 (25)
	5.1.1.6. Living with family	9 (28.13)
	5.1.1.7. Own children/parents/siblings/extended family	2 (6.25)
	Living with roommates/companion	3 (9.38)
	Temporary accommodation	3 (9.38)
**Education (** ***n*** **, %)**		
	School level qualifications (GCSEs, standard or higher grades, GNVQ, A levels, SVQ levels 1 or 2)	12 (27.5)
	Further education Advanced GNVQ, HNC or HND, SVQ levels 3 or 4	7 (21.88)
	Higher education Undergraduate, postgraduate, or PhD degree, SVQ level 5, professional qualification	4 (12.5)
	No qualifications	9 (28.13)
**Psychiatric history**		
5.1.1.8. *Lifetime*		
Hospitalised due to mental health problems **(** ***n*** **, %)**	Yes	17 (53.12)
Number of past hospitalisations for mental health **(mean, SD)**	Of the yes group	1.46 (3.09)
5.1.1.9. *Past 6 months*		
Received treatment for mental health difficulties **(** ***n*** **, %)**	Yes	15 (46.87)
	No	17 (53.12)
	Outpatient psychiatry (CPN, psychiatrist)	5 (15.63)
	GP (Medication management)	2 (6.25)
	Counselling/therapy	3 (9.38)
	Addictions and alcohol groups	1 (3.12)
***Current or past diagnosis* (n, %)***		
**Multiple options allowed for this question*	Mood disorder	27 (84.38)
	Anxiety	12 (37.5)
	Alcohol/substance misuse	10 (31.25)
	Attention-deficit hyperactivity disorder	0
	Conduct problems	6 (18.75)
**Suicide history (** ***n*** **, %)**		
5.1.1.10. *Family history of suicide*		
Family member has died by suicide	Yes	4 (12.5)
Family member has attempted suicide	Yes	10 (31.25)
5.1.1.11. *Lifetime (n %)*		
Has made a suicide attempt	Yes (*n* = 32)	27 (84.38)
Number of suicide attempts **(mean, SD)**	Of the yes group	9.06 (23.98)
Had an interrupted or aborted suicide attempt **(** ***n*** **, %)**	Yes	19 (59.38)
Number of interrupted or aborted suicide attempts **(mean, SD)**	Of yes group (*n* = 31**)	1.13 (11.81)
Engaged in NSSI **(** ***n*** **, %)**	Yes (*n* = 32)	19 (59.38)
Number of NSSI **(mean, SD)**	Of the yes group (*n* = 32)	2.59 (17.62)
Had a preparatory act taken towards a suicide attempt **(** ***n*** **, %)**	Yes (*n* = 32)	11
Number of preparatory acts taken towards a suicide attempt **(mean, SD)**	Of yes group (*n* = 10**)	0.72 (25.31)
5.1.1.12. *Past 3 months*		
Has made a suicide attempt **(** ***n*** **, %)**	Yes (*n* = 32)	30
Number of suicide attempts **(mean, SD)**	Of yes group	1.25 (0.56)
Had an interrupted or aborted suicide attempt **(** ***n*** **, %)**	Yes (*n* = 32)	7 (5.93)
Number of interrupted or aborted suicide attempts **(mean, SD)**	Of yes group (*n* = 7)	0.62 (1.7)
Engaged in NSSI **(** ***n*** **, %)**	Yes (*n* = 32)	9 (28.13)
Number of NSSI incidents **(mean, SD)**	Of the yes group	0.47 (0.88)
Had a preparatory act taken towards a suicide attempt **(** ***n*** **, %)**	Yes (*n* = 32)	9 (28.13)
Number of preparatory acts taken towards a suicide attempt **(mean, SD)**	Of the yes group (*n* = 9)	1.11 (0.37)

## Appendix 7

**Table 11:** Phase II participants’ scores on the psychological measures

Variable	Subcategory	All participants (mean, SD)
ESSI (*n* = 32)		
	Total	21.41 (7.44)
INQ		
	Burdensomeness	33.28 (8.25)
	Thwarted belongingness	27.28 (7.66)
Entrapment scale		
	External entrapment	20.72 (5.71)
	Internal entrapment	24.91 (7.04)
CSSR-S		
	Severity of suicidal Ideation	3.91 (1.69)
	Intensity of Ideation	20.63 (4.21)

*ESSI*, Enrichd Social Support Scale; *INQ*, Interpersonal Needs Questionnaire; *SCRS*, Suicide-Related Coping Scale; *C-SSRS*, Columbia Suicide Severity Rating Scale

## Appendix 8

**Table 12:** Phase III participants’ scores on the psychological measures

Variable	Subcategory	All participants (***N*** = 120)	SPI + TAU (***N*** = 80)	TAU only (***N*** = 40)
**ESSI**				
	Total social support **(mean, SD)** Missing data (*n*)	19.5 (6.4) 3	19.9 (6.2) 3	18.9 (6.7) 3
**INQ**				
	Burdensomeness **(mean, SD)** Missing data (*n*)	31.0 (8.7) 5	30.4 (8.5) 5	32.0 (9.1) 0
	Thwarted belongingness **(mean, SD)**	21.8 (5.7)	21.9 (5.7)	21.6 (5.7)
**Entrapment scale**				
	External entrapment **(mean, SD)** Missing data (*n*)	23.9 (9.6) 1	23.5 (9.4) 1	24.8 (9.9) 0
	Internal entrapment **(mean, SD)** Missing data (*n*)	18.7 (6.2) 1	18.2 (6.0) 1	19.6 (6.6) 0
**SRCS**				
	Total **(mean, SD)** Missing data (*n*)	37.9 (11.8) 1	38.0 (11.1) 1	37.8 (13.2) 0
	Internal coping **(mean, SD)** Missing data (*n*)	14.8 (5.7) 1	14.6 (5.4) 1	15.4 (6.4) 0
	External coping **(mean, SD)** Missing data (*n*)	17.6 (5.5) 1	18.0 (5.3) 1	16.9 (6.0) 0
**CSSR-S**				
	Suicide ideation **(mean, SD)** Missing data (*n*)	3.8 (1.4) 3	3.8 (1.5) 1	3.8 (1.3) 2
	Intensity of ideation **(mean, SD)** Missing data (*n*)	20.7 (4.4) 13	20.6 (4.4) 9	21.0 (4.7) 4

*ESSI*, Enrichd Social Support Scale; *INQ*, Interpersonal Needs Questionnaire; *SRCS*, Suicide-Related Coping Scale; *CSSR-S*, Columbia-Suicide Severity Rating Scale; *SD*, standard deviation

## Appendix 9

**Table 13:** Progression criteria

Criteria	Indicator	Method of assessment	Was this achieved?
1. Were hospital-based study procedures feasible to deliver and acceptable to staff involved (hospital staff onsite and study staff delivering)? *(E.g. referral, recruitment, assessment, SP session delivery)*	Progression to be agreed in conjunction with Trial Steering Committee (TSC)^1^ based on qualitative data captured around experienced and potential barriers to delivery • No current barriers, or those emerging, have been minor, planned for, and overcome in the past during the course of the feasibility study • Some barriers but for which plans have been made/alternatives prepared • Barriers for which no feasible plan or alternative can be offered/developed	Qualitative data collected in SAFETEL intervention provider focus groups, and clinical staff interviews, analysed as part of the process evaluation and reported on to the TSC Barriers identified, and changes made to study protocol as a result will be reported to TSC Given the small number of participants offering qualitative feedback, value will be placed on individual reports of barriers, not simply those barriers that are frequently reported by different participants	Yes The target number of participants was achieved, and all relevant participant medical records were obtained. Feedback from stakeholders indicate that this intervention is acceptable within a hospital setting
2. Were study procedures feasible to deliver and acceptable to participants (including control arm)? *(E.g. recruitment, consent/information given, assessment, safety planning session, follow-up phone calls)*	Progression to be agreed in conjunction with Trial Steering Committee (TSC) based on qualitative data captured around experienced and potential barriers to delivery • No current barriers, or those emerging, have been minor, planned for, and overcome in the past during the course of the feasibility study • Some barriers but for which plans have been made/alternatives prepared • Barriers for which no feasible plan or alternative can be offered/developed	Qualitative data collected in SAFETEL study participant interviews (intervention and control arms) analysed as part of the process evaluation and reported on to the TSC Complaints made by participants or relevant adverse events will be recorded and reported on to TSC Given the small number of participants offering qualitative feedback, value will be placed on individual reports of barriers, not simply those barriers that are frequently reported by different participants	Yes Participant interviewees expressed that their involvement in the study (in both arms) was both acceptable and flexible to their needs. No complaints were made from participants, and all adverse events were documented and reviewed
3. Was it feasible to deliver Safety Plan in the hospital?	**Feasibility of intervention delivery** • Green: > 90% of Safety Plans delivered at hospital • Amber: 60–90% • Red: < 60%	% of safety plans delivered	97.5% (*n* = 78) of participants allocated to the SPI + TAU arm completed the Safety Plan
4. Was it feasible to deliver 1st follow-up phone call attempt within 72 h?	**Was the progression criteria met?** • Green: > 90% of first calls made within 72 h of discharge • Amber: 60–90% • Red: < 60%	**Feasibility of intervention delivery** Research staff attempted to contact 95% of participants in the SPI + TAU arm. Reasons for not attempting contact for the remaining four participants were due to participant unavailability or declining the telephone intervention. In all cases, this was established during baseline contact with the participant	91.3% of 1st follow-up call delivered within 72 h. Additional qualitative data from SAFETEL intervention provider focus groups, risk log, changes to study protocol identifying barriers, and facilitators to implementation reported to TSC
5. Was the target rate of recruitment and retention achieved? *(Are appropriate and effective routes of recruitment available to achieve a powered sample size in a full trial?)*	**Actual recruitment rate vs. target Recruitment rate** • Green: > 80% of participants • Amber: 60–80% • Red: < 60%	**Actual recruitment rate vs. target recruitment rate** Actual participant recruitment rate and target recruitment rate will be measured to support projection of a powered sample for a full trial	Yes The recruitment of 120 participants in Phase III was achieved
6. Was it feasible to attain a minimum dose target required to justify a full trial?	**Adherence rates** • Green: over 80% • Amber: 60–80% inclusive • Red: less than 60%	**Feasibility of attaining minimum dose** % of participants who completed minimum dose participation (i.e. SP + 1 follow-up call)	Yes Over 80% of participants engaged with the minimum dose criteria of this study. Therefore, the outcome of these criteria is ‘green’
7. Was a target rate of completed baseline measures achieved?	**Completion of core measures** • Green: more than 90% data completion • Amber: 70–90% inclusive • Red: less than 70%	**Completion of core measures** • % of participants completed the core questionnaires • % of missing data from completed core questionnaires	Yes A total of 99.99% of all study items were completed by Phase III participants. Therefore, the outcome of this criteria is ‘green’
8. Are identified barriers and challenges to implementation of and adherence to the intervention planned for and surmountable?	Progression to be agreed in conjunction with Trial Steering Committee based on qualitative data captured around experienced and potential barriers to implementation of and adherence to the intervention beyond those already captured in criteria 1 and 2.	Process evaluation report SWOT analysis	No major barriers were identified

Green, very strong indication to proceed. Amber, medium indication to proceed. Discuss with TSC and proceed with identified plan to improve performance on indicator in Phase III trial. Red, indication of doubt as to whether to proceed. Discuss with TSC, and only proceed if other indicators are amber/green, and there is a clear mitigating strategy

## References

[1] Samaritans (2018) Suicide Statistics Report: latest statistics for the UK and Republic of Ireland. Retrieved from: Samaritans: suicide factors and figures. URL: https://www.samaritans.org/scotland/about-samaritans/research-policy/suicide-facts-and-figures/. Date Accessed: 22 Aug 2021

[2] O’Connor RC, Wetherall K, Cleare S, Eschle S, Drummond J, Ferguson E, O’Connor DB, O’Carroll RE (2018). Suicide attempts and non-suicidal self-harm: national prevalence study of young adults. BJPsych Open..

[3] Carroll R, Metcalfe C, Gunnell D (2014). Hospital presenting self-harm and risk of fatal and non-fatal repetition: systematic review and meta-analysis. PLoS One.

[4] National Institute for Health and Care Excellence (2011). Self-harm in over 8s: long-term management. www.nice.org.uk/guidance/cg133 31891461

[5] Mars B, Cornish R, Heron J, Boyd A, Crane C, Hawton K, Lewis G, Tilling K, Macleod J, Gunnell D (2016). Using data linkage to investigate inconsistent reporting of self-harm and questionnaire non-response. Arch Suicide Res.

[6] Turecki G, Brent DA, Gunnell D, O’Connor RC, Oquendo MA, Pirkis J, Stanley BH (2019). Suicide and suicide risk. Nat Rev Dis Prim.

[7] Chung D, Hadzi-Pavlovic D, Wang M, Swaraj S, Olfson M, Large M (2019). Meta-analysis of suicide rates in the first week and the first month after psychiatric hospitalisation. BMJ Open.

[8] Vuagnat A, Jollant F, Abbar M, Hawton K, Quantin C. Recurrence and mortality 1 year after hospital admission for non-fatal self-harm: a nationwide population-based study. Epidemiol Psychiatr Sci. 2020;29. 10.1017/S2045796019000039 PMC8061131 30773154

[9] Stanley B, Brown GK, Brenner LA, Galfalvy HC, Currier GW, Knox KL, Chaudhury SR, Bush AL, Green KL (2018). Comparison of the safety planning intervention with follow-up vs usual care of suicidal patients treated in the emergency department. JAMA Psychiatry.

[10] Craig P, Dieppe P, Macintyre S, Michie S, Nazareth I, Petticrew M (2008). Developing and evaluating complex interventions: the new Medical Research Council guidance. BMJ..

[11] Eldridge SM, Lancaster GA, Campbell MJ, Thabane L, Hopewell S, Coleman CL, Bond CM (2016). Defining feasibility and pilot studies in preparation for randomised controlled trials: development of a conceptual framework. PloS one.

[12] O’Connor RC, Lundy JM, Stewart C, Smillie S, McClelland H, Syrett S, Gavigan M, McConnachie A, Smith M, Smith DJ, Brown GK (2019). SAFETEL randomised controlled feasibility trial of a safety planning intervention with follow-up telephone contact to reduce suicidal behaviour: study protocol. BMJ Open.

[13] Skivington K, Matthews L, Simpson SA, Craig P, Baird J, Blazeby JM, Boyd KA, Craig N, French DP, McIntosh E, Petticrew M, Rycroft-Malone J, White M, Moore L (2021). A new framework for developing and evaluating complex interventions: update of Medical Research Council guidance. BMJ.

[14] Moore GF, Audrey S, Barker M, Bond L, Bonell C, Hardeman W, et al. Process evaluation of complex interventions: Medical Research Council guidance. BMJ. 2015;350. 10.1136/bmj.h1258 PMC4366184 25791983

[15] Brown, G. K., & Stanley, B.. Safety Plan Intervention Rating Scale (SPIRS). Unpublished.

[16] Braun V, Clarke V (2006). Using thematic analysis in psychology. Qual Res Psychol.

[17] Braun V, Clarke V (2013). Successful qualitative research: a practical guide for beginners.

[18] Vaglio J, Conard M, Poston WS, O'Keefe J, Haddock CK, House J, Spertus JA (2004). Testing the performance of the ENRICHD Social Support Instrument in cardiac patients. Health Qual Life Outcomes.

[19] Van Orden KA, Cukrowicz KC, Witte TK, Joiner TE (2012). Thwarted belongingness and perceived burdensomeness: construct validity and psychometric properties of the Interpersonal Needs Questionnaire. Psychol Assess.

[20] Gilbert P, Allan S (1998). The role of defeat and entrapment (arrested flight) in depression: an exploration of an evolutionary view. Psychol Med.

[21] Stanley B, Green KL, Ghahramanlou-Holloway M, Brenner LA, Brown GK (2017). The construct and measurement of suicide-related coping. Psychiatry Res.

[22] Posner K, Brown GK, Stanley B, Brent DA, Yershova KV, Oquendo MA, Currier GW, Melvin GA, Greenhill L, Shen S, Mann JJ (2011). The Columbia–Suicide Severity Rating Scale: initial validity and internal consistency findings from three multisite studies with adolescents and adults. Am J Psychiatry.

[23] O’Connor RC, Kirtley OJ (2018). The integrated motivational–volitional model of suicidal behaviour. Philosoph Transact Royal Society B: Biological Sciences.

[24] Landes SJ, McBain SA, Curran GM (2019). An introduction to effectiveness-implementation hybrid designs. Psychiatry Res.

[25] Milner AJ, Carter G, Pirkis J, Robinson J, Spittal MJ (2015). Letters, green cards, telephone calls and postcards: systematic and meta-analytic review of brief contact interventions for reducing self-harm, suicide attempts and suicide. Brit J Psychiatry.

[26] Doupnik, S.K., Rudd, B., Schmutte,T., Worsley, D., Bowden, C.F., McCarthy, E.F., Eggan, E., Bridge, J.A. & Marcus, S.C. (2020). Association of suicide prevention interventions with subsequent suicide attempts, linkage to follow-up care, and depression symptoms for acute care settings: a systematic review and meta-analysis. JAMA Psychiatry, 77*,* 1021-1030. 10.1001/jamapsychiatry.2020.1586 PMC7301305 32584936

[27] Nuij C, van Ballegooijen W, de Beurs D, Juniar D, Erlangsen A, Portzky G, O’Connor RC, Smit JH, Riper H (2021). Safety planning-type interventions for suicide prevention: meta-analysis. Bri J Psychiatry.

[28] Scotland’s Census (2011). https://www.scotlandscensus.gov.uk/census-results/at-a-glance/ethnicity/ Accessed 18 Jan 2022.

